# The Washing of New-Born Infants

**Published:** 1911-12-23

**Authors:** J. C. McWalter

**Affiliations:** Dublin


### The Editor's Letter Box.

### THE WASHING OF NEW-BORN INFANTS.

*To the Editor of* The Hospital.

Sir,—Scientific heresy is the beginning of wisdom, and at the risk therefore of being burnt at the stake of ridicule as a dirt-worshipper—I ask why wash the child? By the child I mean the infant—anything under 24 hours old.

What happens? No sooner has the little greasy mass of flesh, lately ejected into this world of sorrow, had its navel string tied—no sooner has it started on independent existence—than a couple of harpies proceed to remove its soothing garment of balmy *vermix caseosa.* The unfortunate infant howls for mercy, but all in vain—the flaying process goes on till finally the intensely sensitive skin is excoriated with soap and water, and the child dozes off—exhausted. Nature does nothing in vain. Is a fœtus enveloped in this fatty covering merely as a crude joke?

We are told that it probably prevents the absorption of the amniotic fluid and assists the delivery of the child. Granted. At least, it has some important physiological function to discharge. Why, therefore, assume that it ceases to discharge useful functions once the child is born? May it not be designed to inure the infant to the extra-uterine variations of temperature? Figures quoted by Jellett seem to show that new-born infants not subjected to daily bathing have a much better chance of life than those who are immersed in water every day. If this be so it seems reasonable to withhold the proto-ablution for a few hours.

For centuries the umbilical cord was tied and cut the moment the child was born. Hundreds of thousands of lives were lost by this unnatural and unseemly haste. Nobody but an ignorant fool now cuts the cord till it stops pulsating. Anyone can see the fœtus improving with every heart beat so long as the cord is uncut. Meddlesome midwifery is bad midwifery, but yet we persist in meddling with the child's covering. I suggest we ought to give Nature a chance.—Yours, truly,

J. C. McWalter,

Dublin, December 19. M.A., LL.B., M.D. Brux.

